# Supplementary material for: Phosphorylation-induced SUMOylation promotes Ulk4 condensation at ciliary tip to transduce Hedgehog signal
Source: bioRxiv. 2024 Nov 22:2024.09.19.613872. Preprint. [Version 2] doi: 10.1101/2024.09.19.613872 (PMC11601359; doi:10.1101/2024.09.19.613872)
Supplement: Supplement 1 [file NIHPP2024.09.19.613872v2-supplement-1.pdf]

bioRxiv preprint doi: https://doi.org/10.1101/2024.09.19.613872; this version posted November 22, 2024. The copyright holder for this preprint (which was not certified by peer review) is the author/funder, who has granted bioRxiv a license to display the preprint in perpetuity. It is made available under aCC-BY 4.0 International license.

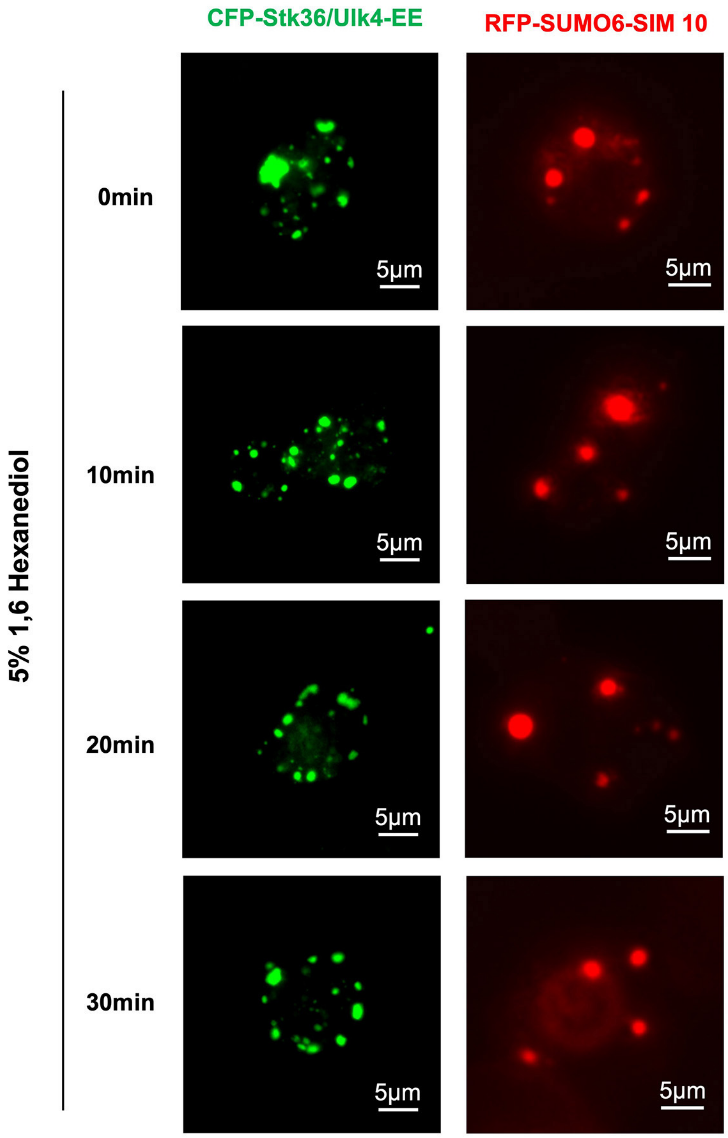

Fig. S1. Stk36/Ulk4 condensates are resistant to 1, 6-hexanediol

Representative images of HEK293T cells expressing the indicated constructs before and after treatment with 5% 1, 6-hexanediol for 10, 20, and 30 minutes.

Scale bars are 5 µm.

bioRxiv preprint doi: https://doi.org/10.1101/2024.09.19.613872; this version posted November 22, 2024. The copyright holder for this preprint (which was not certified by peer review) is the author/funder, who has granted bioRxiv a license to display the preprint in perpetuity. It is made available under aCC-BY 4.0 International license.

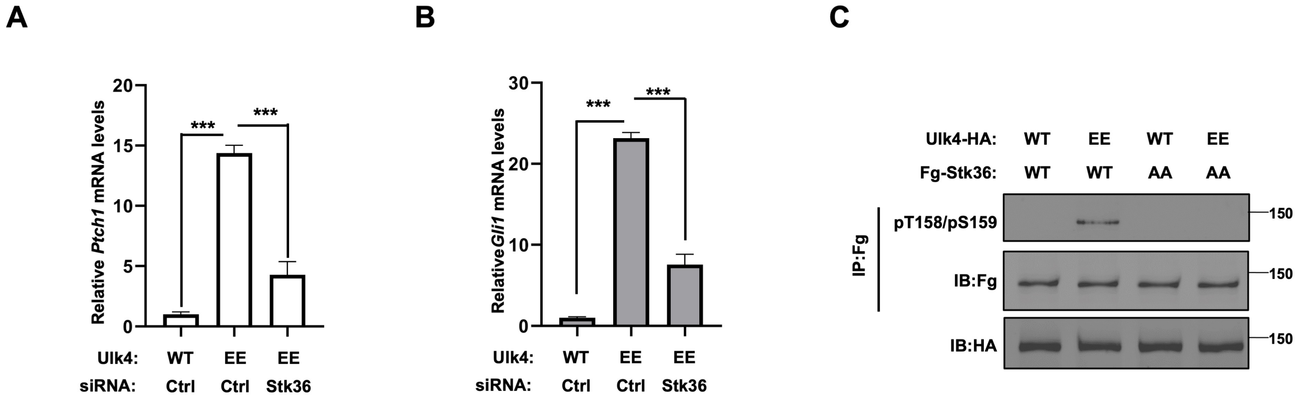

Fig. S2. The constitutive pathway activity of Ulk4-EE depends on Stk36

(A, B) Relative *Ptch1* (A) and *Gli1* (B) mRNA levels in NIH3T3 cells infected with the indicated Ulk4 lentiviral constructs and treated with control (Ctrl) or Stk36 siRNA.

(C) Western blot analysis of Stk36 phosphorylation on pT158/pS159 in NIH3T3 cells co-infected with the indicated Ulk4 and Stk36 lentiviral constructs.
